# Role of microplastics in the survival and antimicrobial susceptibility of *Campylobacter jejuni*

**DOI:** 10.3389/fmicb.2025.1717297

**Published:** 2026-01-05

**Authors:** Irene Ortega-Sanz, Andreja Rajkovic

**Affiliations:** Department of Food Technology, Safety and Health, Faculty of Bioscience Engineering, Ghent University, Ghent, Belgium

**Keywords:** biofilm formation, Etest, whole-genome sequencing, phenomics, environmental microbiology, food safety, public health, comparative genomics

## Abstract

Microplastics (MPs) are a global concern due to their persistence in the environment and capacity to carry pollutants and pathogenic microorganisms. Given recent evidence on the co-occurrence of MPs and *Campylobacter* spp., the leading cause of foodborne gastrointestinal infections worldwide, this study investigates the role of MPs in *Campylobacter jejuni* contamination and their impact on antimicrobial susceptibility. The potential of five *C. jejuni* isolates from different origin (poultry, water, and human) to form biofilms on MPs over time (24 h, 48 h, and 72 h) was evaluated using traditional culture-dependent methods, including the reference strain *C. jejuni* subsp. *jejuni* strain NCTC 11168. The effect of MPs on the antimicrobial susceptibility of *C. jejuni* cells detached from MP-associated biofilms was also assessed using Etest strips. To comprehensively understand the interactions between the MPs and the bacteria, whole-genome sequencing was performed to explore the presence of adherence and biofilm-associated genes, as well as antibiotic resistance genes. The strains rapidly colonized the MPs within 24 h, exhibiting varying attachment densities over time ranging from 1.09 to 5.78 log CFU/MP, with strain NCTC 11168 identified as the strongest biofilm former overall. Furthermore, the abundance of adherence and biofilm formation genes was consistent with their abilities to form biofilm on MPs. The gene *peb3* played a critical role in determining biofilm formation levels on MPs, while specific combinations of *capA*, *capB*, *cj1725*, and *porA* were linked to enhanced biofilm development. Similarly, the presence of antibiotic resistance determinants aligned with the phenotypic resistance, and only one strain (ST-6209/CC464) exhibited resistance to ciprofloxacin, nalidixic acid, and tetracycline. Notably, antimicrobial susceptibility of cells detached from MP-biofilms was increased by up to 4.6 log_2_-fold compared to planktonic counterparts. The findings indicate that MPs can facilitate the persistence of *C. jejuni* in the environment while simultaneously increasing their susceptibility to antibiotics. Further research with larger cohorts is needed to validate these preliminary observations in order to support the development of effective policies addressing MP pollution and food safety.

## 1 Introduction

Microplastics (MPs) represent an emerging threat to human health and planetary sustainability. Defined as plastic particles between 1 μm and 5 mm, they originate either as primary MPs or through the fragmentation of larger plastics. Their widespread occurrence, exceptional environmental persistence, and high potential to transport and release toxic additives, hazardous chemicals, and co-occurring pollutants underscore their significance as a global concern (Tavelli et al., 2022; Ziani et al., 2023). Although the interaction between MPs and foodborne pathogens has received little attention, previous studies have detected the presence of *Campylobacter* spp. —the leading bacterial cause of foodborne illness globally—on MPs (Kaakoush et al., 2015; Ortega-Sanz and Rajkovic, 2025). However, the extent of this pathogen’s association with MPs and the potential effects on its physiology and behavior remains largely unexplored. Hence, there is an urgent task of understanding how MPs interact with the bacteria to further measure the impact of MPs on their survival, persistence and pathogenicity in the environment and along the poultry supply chain in particular, where the bacteria are highly persistent (Ortega-Sanz et al., 2025).

Among *Campylobacter* species, *Campylobacter jejuni* poses a particularly significant food safety risk, especially in the poultry industry, where it can spread via carcass cross-contamination. In 2023, 148,181 confirmed cases of human campylobacteriosis were reported in the EU, resulting in an estimated public health burden of €2.4 billion annually (EFSA, 2011; EFSA and ECDC, 2024). Despite its public health importance, the role of MPs in *Campylobacter* transmission remains uncertain, although research suggests that environmental factors are key in severe complications of campylobacteriosis (Ortega-Sanz et al., 2023b). MPs provide physical support to *C. jejuni* for biofilm formation, protecting them from extreme conditions and facilitating their persistence in the environment. Likewise, bacteria can detach from the biofilm and disperse, colonizing new niches (Purevdorj-Gage et al., 2005). These biofilm formation and biofilm dispersal phenomena could explain why *Campylobacter* are able to survive and persist in the environment, despite being highly sensitive to atmospheric oxygen concentrations (Kaakoush et al., 2007). Indeed, biofilm formation plays an important role in the dissemination of *Campylobacter* from avian reservoirs to humans, thus increasing its potential to cause disease (Tram et al., 2020). Hence, MPs pose a significant challenge to the poultry industry as they could become a source of *C. jejuni* and promote cross-contamination events. In food processing facilities, plastic materials like polyethylene (PE), polypropylene (PP), and polyethylene terephthalate (PET) constitute a potential source of MPs, which provide an ideal surface for *C. jejuni* biofilm formation. Therefore, understanding the role of MPs in the transmission of the pathogen is critical for the development of efficient control strategies against both MP and *Campylobacter* contamination.

During biofilm formation, the extracellular matrix can impede antimicrobial penetration, leading to increased *C. jejuni* resistance compared to planktonic cells (Wagle et al., 2019; Ramić et al., 2021). For instance, resistance to gentamicin in biofilm-associated *C. jejuni* was observed to be as much as 32 times greater than in their planktonic counterparts (Malik et al., 2017). Whether MPs can induce a similar effect is still unexplored. Moreover, exposure of *C. jejuni* biofilm cells to sublethal antibiotic levels results in constant selective pressure that triggers adaptive responses in the bacteria, including the emergence of spontaneous mutations conferring antibiotic resistance, gene recombination, and horizontal gene transfer (HGT) events at higher rates (up to 17.5-fold) than planktonic cells (Bae et al., 2014; Svensson et al., 2014; Ma et al., 2021). Thus, biofilm formation by *C. jejuni* plays a key role in AMR development. However, it remains unclear whether these mechanisms also contribute to AMR in *C. jejuni* cells adhered to MPs. Consequently, it is crucial to investigate how the presence of MPs affects antibiotic resistance in *C. jejuni*, especially given evidence that MPs can promote multidrug resistance in other foodborne pathogens, such as *E. coli* (Gross et al., 2025).

This study evaluates the role of MPs as vehicles of *C. jejuni* contamination and their impact on antibiotic resistance. This pioneering research offers insight into the mechanisms supporting the survival of *C. jejuni* in the environment through the quantification of bacterial cells adhered to microplastics and their potential effect on antibiotic resistance. Understanding the interaction between *C. jejuni* and MPs will help to design targeted control measures to reduce environmental contamination and limit AMR development.

## 2 Materials and methods

### 2.1 Bacterial strains and growth conditions

A total of five *Campylobacter jejuni* strains were selected from the bacterial strain collection of the Research Unit Food Microbiology and Food Preservation (FMFP) at Ghent University (Belgium). The strains were selected to represent diverse sources within the poultry supply chain as well as clinical samples, including the well-characterized *C. jejuni* subsp. *jejuni* strain NCTC 11168. Detailed information regarding these isolates is presented in Table 1.

**Table 1:** Information of the *C. jejuni* strains used in this study.

Strain	Origin	Country	References
CN 30	Human	The Netherlands	Cools et al. (2003)
KC 47.1	Poultry	Belgium	Cools et al. (2003)
NCTC 11168	Human (faeces)	The Netherlands	FMFP culture collection
RIZA 192	Water	The Netherlands	Cools et al. (2003)
T 84 nr 14	Frozen chicken (84 days)	Belgium	Sampers et al. (2010)

FMFP, Food Microbiology and Food Preservation Research Unit at Ghent University (Belgium).

Stock cultures in Brain Heart Infusion Broth (BHI; Oxoid) with 20% glycerol were stored at −80 °C. Before use in assays, *C. jejuni* strains were grown on blood agar (BA) plates containing Nutrient Broth No. 2 (Oxoid, Basingstoke, UK) with 1.5% bacteriological agar (Oxoid) supplemented with 5% (v/v) defibrinated sheep blood (Oxoid) under microaerophilic conditions at 37 °C for 48 h. Microaerophilic conditions were generated with a CampyGen™ sachet (Thermo Fischer Scientific, Paisley, UK).

### 2.2 Quantification of biofilm formation on microplastics

Microplastic discs (5 mm) were prepared from biaxially oriented polyethylene terephthalate (BOPET) heat seal film used for food packaging (thickness: 30.5 μm; density: 1.40 g/cm^3^). Prior to assays, MPs were prepared, sterilized with 70% ethanol for 5 min, and allowed to dry overnight at room temperature. In an untreated Falcon™ 24-well polystyrene microplate (F-bottom; Corning Inc., Corning, NY, USA), one dried, sterile disc was placed per well. Bacterial inocula were prepared in Mueller-Hinton broth (MHB) from 24-h bacterial lawns grown on BA plates and adjusted to an optical density of 0.1 at 600 nm (approximately corresponding to 10^7^ CFU/mL), and 500 μL of the suspension were added per well. Positive planktonic bacterial controls consisted of bacterial cultures in MHB without microplastic discs (i.e., free-living bacteria in liquid medium), whereas negative controls consisted of wells containing only MHB or MHB with microplastic discs (without bacteria). The microplates were incubated under microaerobiosis at 37 °C for 72 h.

Every 24 h, the planktonic bacteria from the wells with and without discs (referred to as “planktonic (MP)” and “planktonic (no-MP)” in the Results section, respectively) were collected for bacterial enumeration on BA, and the discs were carefully washed in sterile distilled water to remove non-adherent bacteria. Each disc was then sonicated in 500 μL of peptone saline solution (PPS; 1 g/L neutralized bacteriological peptone [LP0034, Oxoid] and 8.5 g/L NaCl [CL00.1429.5000-5KG, AnalytiChem Belgium NV, Zedelgem, Belgium]) using a water bath sonicator (37 kHz; Elmasonic S 30 H, Elma Schmidbauer Gmbh, Baden-Württemberg, Germany) for 5 min to detach biofilm-forming bacteria. During sonication, the temperature of the bacterial suspension increased by approximately 9 °C, elevating the initial temperature of 21.1–21.6 °C. The sonication conditions applied were confirmed not to affect the viability of the bacterial strains as no difference was observed in number of CFU compared to cultures that were not sonicated (data not shown). After sonication, serial dilutions of the bacterial cultures were performed in PPS for bacterial enumeration on BA. Bacterial concentrations were expressed as log CFU/MP for sessile cells on MP discs and log CFU/mL for planktonic cells in suspension. The experiment was performed in triplicate and repeated three independent times (*n* = 3).

### 2.3 Antimicrobial susceptibility of biofilm-derived bacteria from MPs

Antimicrobial susceptibility of bacterial cells detached from MP-associated biofilms was assessed using the Etest^®^ method and compared with that of planktonic bacteria. Six antibiotics belonging to four different classes were tested. Two (fluoro)quinolones: ciprofloxacin (CIP; 0.002–32 mg/L) and nalidixic acid (NAL; 0.016–256 mg/L); two macrolides: erythromycin (ERY; 0.016–256 mg/L) and azithromycin (AZM; 0.016–256 mg/L); one aminoglycoside: gentamicin (GEN; 0.016–256 mg/L); and tetracycline (TET; 0.016–256 mg/L). Biofilms were allowed to form on MPs following the previously described procedure. However, any strain forming biofilm below 3.5 log CFU/MP was excluded from this assay. After 72 h of biofilm formation, biofilm bacteria were harvested by first gently submerging the discs in sterile distilled water to remove non-attached bacterial cells, followed by sonication in 500 μL of MHB for 5 min to detach the adhered bacteria. Planktonic bacteria were prepared from overnight cultures by inoculating a single fresh colony from a BA plate into 5 mL MHB, further incubated under microaerobic conditions at 37 °C for 24 h. Bacterial suspensions from both biofilm and planktonic cultures, without any cell density adjustment, were spread onto blood agar (BA) plates with a thickness of 4.0 ± 0.5 mm and allowed to dry under sterile conditions for 15 min. We aimed to obtain uniform bacterial lawns for reliable MIC values, rather than relying on identical initial bacterial turbidities, since identical turbidity readings do not necessarily correspond to the correct number of viable cells required for confluent growth. Immediately after, antibiotic gradient Etest strips (bioMérieux, 2019) were placed on the agar plates at equal distances using sterile tweezers. To facilitate interpretation, no more than four Etest strips were applied per 150 mm plate, and only one strip per 90 mm plate, as recommended by the manufacturer (bioMérieux, 2019). Each strip was gently pressed at the top from the lowest to the highest concentration using sterile tweezers to ensure proper contact with the agar surface. Plates were incubated upside up (lid up) at 37 °C for 48 h in microaerophilic conditions according to the application guide of the manufacturer. The minimum inhibitory concentration (MIC) was determined at the intersection of the gradient antibiotic strip and the zone of inhibition. When the intersection occurred between two serial dilutions, the highest dilution was recorded as the MIC. Strains were classified as susceptible (S) or resistant (R) based on epidemiological cutoff values (ECOFFs) according to the European Committee on Antimicrobial Susceptibility Testing (EUCAST) (2025). Experiments were repeated three independent times (*n* = 3), expressing the MICs as the median of the 3 MIC values obtained for each antibiotic.

### 2.4 Genomic DNA extraction, whole genome sequencing (WGS) and assembly

Genomic DNA extraction was performed using the DNeasy PowerSoil Pro Kit (Qiagen, Antwerp, Belgium) following the manufacturer’s instructions. Specifically, all centrifugation steps were performed at 13,000 rpm, and DNA was eluted in 100 μL of Solution C6. Concentration and purity of the extracted DNA were measured using the Qubit 4 fluorometer (Thermo Fischer Scientific, Paisley, UK) and BioDrop μLite+ spectrophotometer (Biochrom, Gründau, Germany), respectively. Library preparation and sequencing was carried out by Novogene (Cambridge, UK). PCR-free libraries were constructed with the Novogene NGS DNA Library Prep Set kit (Cat No. PT004), in which the gDNA was randomly sheared into shorter fragments, followed by end repair, A-tailing, and ligation with Illumina adapters. Further library controls included Qubit fluorometry and qPCR for library quantification, and fragment analysis using the Agilent 2100 Bioanalyzer to assess size distribution. Fragments between 320 and 350 bp were selected and purified prior to sequencing. Quantified libraries were pooled and sequenced on the NovaSeq X Plus platform (Illumina), generating 2 × 150 bp paired-end reads with a minimum yield of 1 GB of raw data per sample.

The CamPype pipeline (Ortega-Sanz et al., 2023a) was used to filter and assemble the raw reads. Briefly, read quality was assessed by Fastqc v0.12.1 (S. Andrews^1^) and quality filtering was performed using Trimmomatic v0.39 (Bolger et al., 2014). Adapters and other Illumina technical sequences were removed from the read sequences using the ILLUMINACLIP option (2:30:10) using the adapters_and_sequences.fa file available at https://github.com/JoseBarbero/CamPype/tree/master/reference_files (accessed September 18, 2025). Low-quality bases were trimmed from the leading and trailing ends of reads if their quality was below 30 (LEADING:30; TRAILING:30). Additionally, a 10-base sliding window was applied, cutting reads when the average quality within the window fell below 30 (SLIDINGWINDOW:10:30). Reads were then *de novo* assembled by SPAdes v4.1.0 using the isolate mode (Prjibelski et al., 2020), incorporating the reference genome of strain NCTC 11168 (NCBI Ref Seq accession NC_002163.1) as trusted contigs solely for this strain. Contigs shorter than 500 bp were removed from the assemblies (Ortega-Sanz et al., 2025). Assembly quality and completeness were assessed with QUAST v5.2.0 (Mikheenko et al., 2018) and CheckM2 v1.1.0 (using the uniref100.KO.1.dmnd database; Chklovski et al., 2023), respectively. Contigs of draft genomes were reordered against the reference genome *C. jejuni* NCTC 11168 (NC_002163.1) using progressiveMAUVE v2.4.0 (Darling et al., 2010).

### 2.5 Characterization of *C. jejuni* genomes and identification of biofilm-associated genes and antibiotic resistance determinants

To assess the similarity between strains, the whole-genome Average Nucleotide Identity (ANI) was calculated for every genome pair by fastANI v1.34 (Jain et al., 2018). The pairwise ANI values were converted into a distance matrix using the pairwise_identities_to_distance_matrix.py script available at https://github.com/rrwick/Bacsort (accessed September 18, 2025). Then, this matrix was used to compute a BIONJ tree (Gascuel, 1997; Lees et al., 2018) using the R package ape v5.8 (Paradis and Schliep, 2019). The PubMLST *Campylobacter jejuni*/*coli* MLST scheme was used for genotypic sequence typing,^2^ assigning MLST profiles to each assembled genome and subsequently grouping them into Clonal Complexes (CCs) (Jolley et al., 2018). Genomes were annotated by Bakta v1.11.4 (Schwengers et al., 2021) using the light database v6.0 and the curated GenBank annotation from the reference genome *C. jejuni* NCTC 11168 (NC_002163.1) to guide the annotation using default thresholds of 90, 80 and 80% for minimal identity, query and subject coverage, respectively.

Genomes were screened for the presence/absence of adherence and biofilm-associated genes using BLASTN v2.16.0 (Camacho et al., 2009). The in-house database v1 (Ortega-Sanz, 2024; Ortega-Sanz et al., 2025), comprising 14 *C. jejuni* adherence genes and 16 *C. jejuni* biofilm formation genes (Supplementary Table 1), was chosen over VFDB (Chen et al., 2016) because only seven genes associated with *C. jejuni* adherence and biofilm formation (*cadF*, *flpA*, *flgD*, *jlpA*, *pebA*, *porA*, and *peb4*) were identified in the latter. The 30 gene sequences were obtained from the in-house database v1 available on Zenodo.^3^ A gene was considered present if it showed at least 80% nucleotide identity and 80% coverage, consistent with the default thresholds applied in ABRicate v1.0.1 (T. Seemann^4^). The same criteria were applied for detecting known acquired antibiotic resistance genes using ABRicate against the databases ARG-ANNOT (Gupta et al., 2014), CARD (Comprehensive Antibiotic Resistance Database) (McArthur et al., 2013), ResFinder (Zankari et al., 2012), Megares (Lakin et al., 2017) and NCBI Bacterial Antimicrobial Resistance Reference Gene Database (NDARO).^5^ In addition, known point mutations conferring antibiotic resistance were identified by AMRFinderPlus v4.0.23 (Feldgarden et al., 2021) with the database for *Campylobacter* species, applying the previously established identity and coverage thresholds. PlasmidFinder v2.1 (Carattoli et al., 2014) was used to detect plasmid replicons in contigs.

### 2.6 Statistical analysis

Statistical analysis was carried out in R v4.4.0 (R Core Team, 2024). The ability of the different *C. jejuni* strains to form biofilm over time was determined using two-way analysis of variance (ANOVA), which also included the interaction between strain and incubation time. Assumptions of normality and homogeneity of variance were assessed using the Shapiro–Wilk and Levene’s tests, respectively. When the assumptions of ANOVA were violated, the non-parametric Aligned Rank Transform (ART) ANOVA was applied. Differences between means from independent experiments (*n* = 3) were determined using the Tukey method. Regarding antimicrobial susceptibility testing, since MIC values are measured on a discontinuous scale (discrete quantitative variable), differences in median MIC values between planktonic cells and cells detached from biofilms (unpaired data) were evaluated using a non-parametric Mann–Whitney test (*n* = 3) with the exact distribution implemented in the coin library (v1.4-2) (Hothorn et al., 2008). Significance level was established at 95%.

## 3 Results

### 3.1 Dynamics of *Campylobacter jejuni* biofilm formation on microplastics

**Figure 1:**
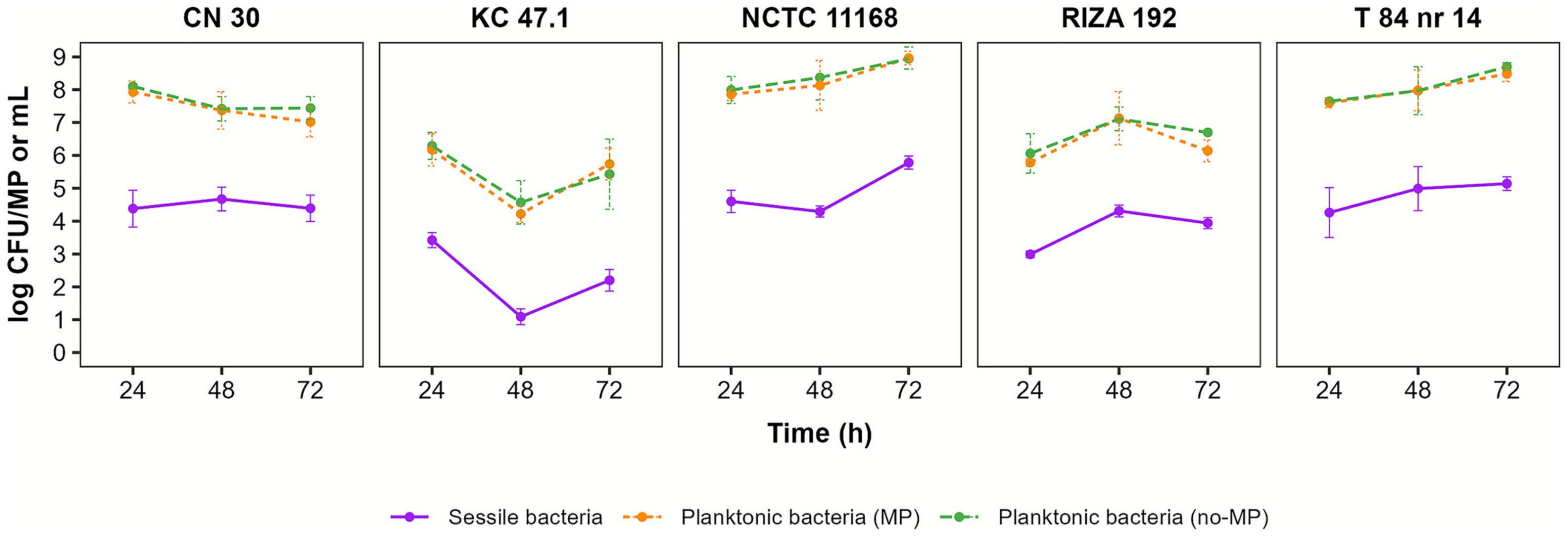
Biofilm development of *Campylobacter jejuni* on microplastics over time at 37 °C under microaerobic conditions. Bacterial counts for sessile cells are expressed as log CFU/MP, while planktonic cells are expressed as log CFU/mL. Data represent the mean ± standard deviation of three independent experiments (*n* = 3). Statistical significance is shown in Supplementary Tables 2, 3. Detection limits of experiment were 0.7 log CFU/MP and 1 log CFU/mL.

Biofilm formation on microplastics (MPs) was significantly influenced by the *C. jejuni* strain, the incubation time, and the interaction between these factors (*p* < 0.05) (Figure 1, Supplementary Table 2). Within 24 h, the strains had rapidly colonized the MPs, with attachment densities varying among them. At this point, strain NCTC 11168 exhibited the highest biofilm density with 4.60 ± 0.34 log CFU/MP, equivalent to 3.31 ± 0.48 log CFU/mm^2^, corresponding to the estimated average surface area of the plastic discs (19.63 mm^2^). Since then, adherence to the MP discs significantly changed over time, except CN 30 and T 84 nr 14, with different dynamic patterns across strains (Supplementary Table 3). Strain RIZA 192 showed significantly elevated biofilm levels at 48 h compared to 24 h, while strain KC 47.1 produced the highest biofilm at 24 h, followed by a significant reduction thereafter. At 72 h, only NCTC 11168 significantly increased its adherence to MPs, whereas KC 47.1 showed a significant reduction compared to initial levels at 24 h. Overall, the highest biofilm formation level was significantly produced by strain NCTC 11168 at 72 h when 5.78 ± 0.20 log CFU/MP were detected, equivalent to 4.44 ± 0.31 log CFU/mm^2^, followed by strain T 84 nr 14 at 48 h and 72 h (Supplementary Table 2). In contrast, strains KC 47.1 and RIZA 192 showed the weakest biofilm formation, particularly KC 47.1 at 48 h (1.09 ± 0.24 log CFU/MP). These two strains also exhibited biofilm formation dynamics following a similar trend to their planktonic growth (Figure 1). In addition, planktonic bacterial growth showed minimal differences in the presence or absence of MPs across all strains. However, their abilities to proliferate in liquid medium differed. While the abundance of strains CN 30, NCTC 11168, and T 84 nr 14 ranged from 7.02 to 8.96 log CFU/mL over time, strains KC 47.1 and RIZA 192 varied between 4.22 and 7.13 log CFU/mL (Supplementary Table 3).

### 3.2 Antibiotic susceptibility of *Campylobacter jejuni* biofilm-derived cells

**Figure 2:**
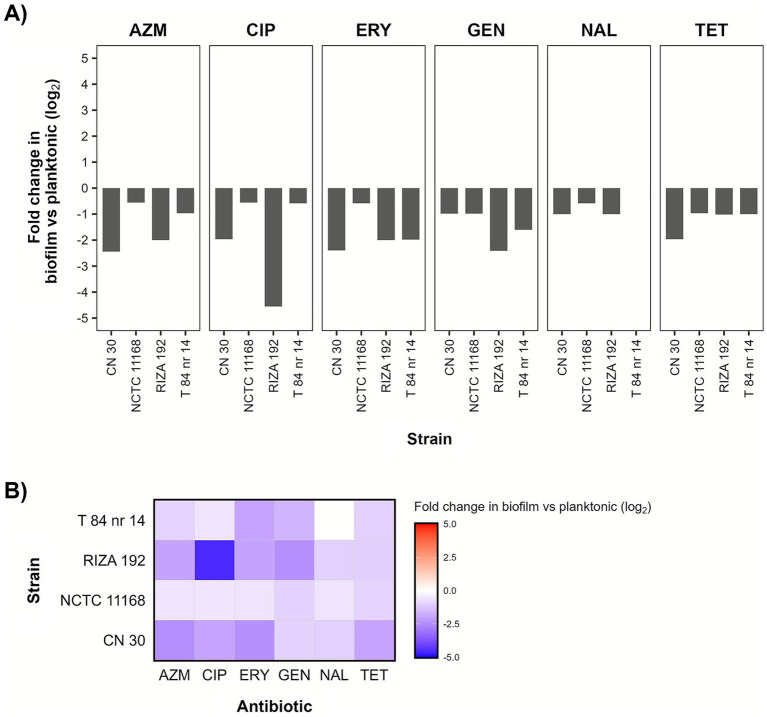
Comparison of antibiotic susceptibility in *Campylobacter jejuni* between detached biofilm and planktonic cells determined by Etest. **(A)** Bar plot showing the log_2_ fold change in median minimum inhibitory concentration (MIC) values between cells detached from MP-associated biofilms and planktonic cells for each combination of strain and antibiotic across three independent experiments (*n* = 3; unpaired data). The log_2_ fold change for strain T84 nr 14 against NAL could not be determined, as the MIC values exceeded the upper detection limit of the Etest strip. No significant differences in MICs were found between planktonic and MP-detached cells (Mann–Whitney *U* test, *p* > 0.05). **(B)** Heatmap of the log_2_ fold change (median MIC detached biofilm cells/median MIC planktonic cells). White squares indicate equal median MIC values, while blue squares indicate lower median MIC values in cells detached from MP-biofilms (greater susceptibility). AZM, azithromycin; CIP, ciprofloxacin; ERY, erythromycin; GEN, gentamicin; NAL, nalidixic acid; TET, tetracycline.

The *C. jejuni* strains exhibited varying susceptibility levels to antibiotics. Only strain T 84 nr 14 exhibited resistance to antibiotics according to ECOFFs, including ciprofloxacin (CIP), nalidixic acid (NAL) and tetracycline (TET) (Table 2). The remaining antibiotics tested, including azithromycin (AZM), erythromycin (ERY) and gentamicin (GEN), were effective, with all strains showing susceptibility. Similar behaviours were observed in the cells detached from biofilms formed on MPs. However, the biofilm-derived bacteria exhibited a notable increase in susceptibility to all antibiotics compared with their planktonic counterparts, despite no statistically significant differences in MIC values were observed for any strain-antibiotic combination (Mann–Whitney U test, *p* > 0.05) (Figure 2). Strain CN 30 displayed the highest increase in antibiotic susceptibility against AZM, ERY and TET among the cells detached from MP-associated biofilms, while strain RIZA 192 for CIP and GEN. Of these, the increase in antibiotic susceptibility of RIZA 192 to CIP exceeded a fourfold change (4.6 log_2_-fold). In contrast, strain NCTC 11168 showed the smallest increase in susceptibility to all antibiotics among cells detached from MPs compared with the planktonic state, except for NAL, where strain T 84 nr 14 showed no lifestyle-related change in resistance.

**Table 2:** Antibiotic susceptibility of planktonic and biofilm-detached *C. jejuni* strains from MPs determined by Etest with ECOFF susceptibility interpretation.

Lifestyle	Strain	AZM	CIP	ERY	GEN	NAL	TET
Planktonic	CN 30	0.125 (S*)	0.125 (S)	1 (S)	0.75 (S)	3 (S)	0.25 (S)
	KC 47.1	0.094 (S)	0.064 (S)	0.75 (S)	0.5 (S)	3 (S)	0.25 (S)
	NCTC 11168	0.094 (S)	0.094 (S)	0.75 (S)	0.75 (S)	3 (S)	0.125 (S)
	RIZA 192	0.064 (S)	0.047 (S)	0.5 (S)	0.5 (S)	1 (S)	0.19 (S)
	T 84 nr 14	0.125 (S)	6 (R)	0.75 (S)	0.38 (S)	>256 (R)	64 (R)
Detached biofilm	CN 30	0.023 (S)	0.032 (S)	0.19 (S)	0.38 (S)	1.5 (S)	0.064 (S)
	KC 47.1	ND^‡^	ND	ND	ND	ND	ND
	NCTC 11168	0.064 (S)	0.064 (S)	0.5 (S)	0.38 (S)	2 (S)	0.064 (S)
	RIZA 192	0.016 (S)	0.002 (S)	0.125 (S)	0.094 (S)	0.5 (S)	0.094 (S)
	T 84 nr 14	0.064 (S)	4 (R)	0.19 (S)	0.125 (S)	>256 (R)	32 (R)

Values show the median minimum inhibitory concentration (MIC) value (mg/L) across three replicates (*n* = 3) (see Supplementary Table 4 for replicate measurements). *Susceptibility interpretation for *C. jejuni* epidemiological cutoff values (ECOFFs): AZM (≥0.25 mg/L), CIP (≥0.5 mg/L), ERY (≥4 mg/L), GEN (≥2 mg/L), NAL (≥16 mg/L), TET (≥1 mg/L); R, resistant; S, susceptible. ^‡^MIC could not be determined (ND, not determined) due to low biofilm formation ability (< 3.5 log CFU/MP), that prevented sufficient agar surface coverage for accurate MIC measurement. AZM, azithromycin; CIP, ciprofloxacin; ERY, erythromycin; GEN, gentamicin; NAL, nalidixic acid; TET, tetracycline.

### 3.3 Genome characterization of the *Campylobacter jejuni* strains

**Figure 3:**
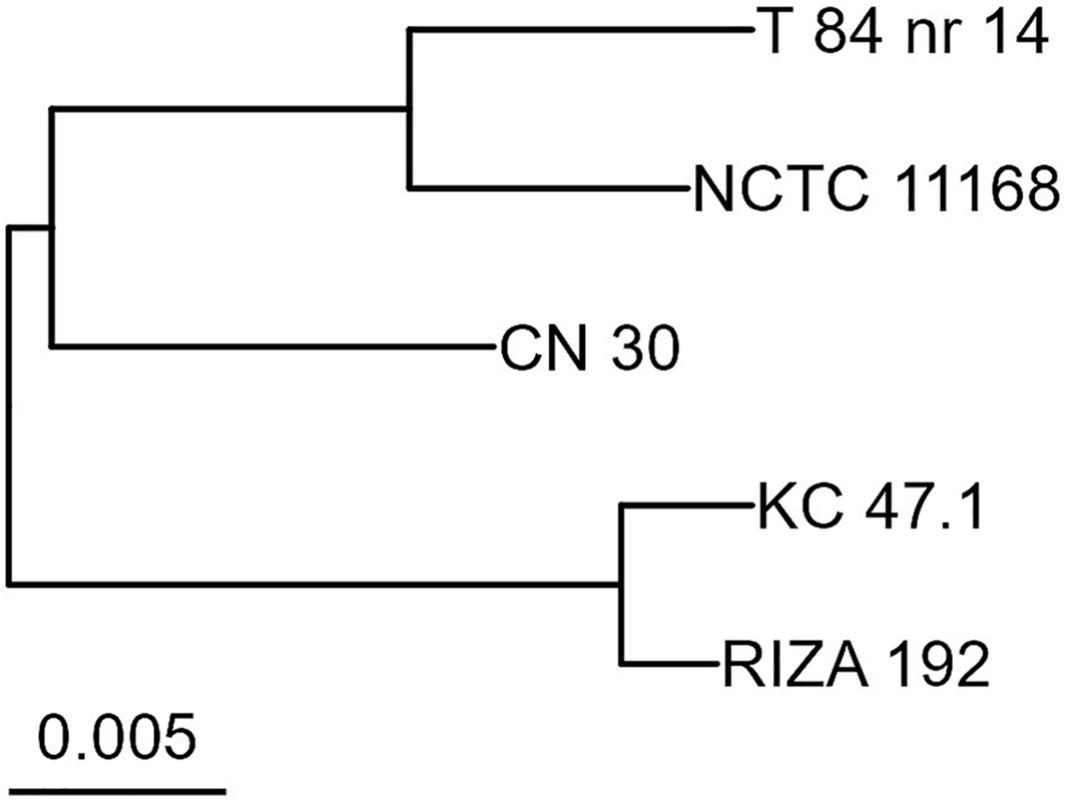
BIONJ tree of the *C. jejuni* strains based on the ANI values showing their relatedness.

A total of 36,474,270 reads were generated from sequencing the five isolates, of which 99.20% (36,182,284) had a quality score of 30 or higher. After filtering, 98.7% of the reads (36,011,884) remained, all maintaining a Q30 or higher quality. The five draft genomes were assembled into 1–76 contigs with a genome length ranging from 1.61 to 1.79 Mbp and a coverage between 491X and 742X (Table 3). The genomes showed ≥99.9% completeness and contained between 1,661 and 1,842 genes. The ANI values among the five isolates ranged between 96.49% and 99.48%, with strains KC 47.1 and RIZA 192 forming a distinct cluster, while strain T 84 nr 14 was more closely related to NCTC 11168 than to CN 30 (Figure 3).

**Table 3:** General genomic characteristics of the *Campylobacter jejuni* isolates analyzed in this study.

Analysis	Feature	CN 30	KC 47.1	NCTC 11168	RIZA 192	T 84 nr 14
Assembly	Completeness (%)	99.99	99.98	99.98	99.98	100
	Contigs	24	76	1	54	33
	Genome length (bp)	1,612,344	1,791,969	1,635,582	1,785,651	1,736,800
	Genome coverage (X)*	742	610	716	577	491
	G + C content (%)	30.45	30.04	30.51	30.07	30.25
	N50	184,176	115,296	153,957	108,410	174,996
Multilocus sequence typing (MLST)	Sequence Type (ST)	ST-11	ST-4467	ST-43	ST-680	ST-6209
	Clonal complex (CC)	CC45	Unknown	CC21	Unknown	CC464
Annotation	Coding DNA sequences (CDS)	1,620	1,840	1,661	1,842	1,793
	CDS with assigned functions	1,550	1,642	1,589	1,648	1,620
	Coding density (annotated bases/genome length)	94.4	91.7	95.2	92.7	93.6
	rRNA	3	3	6	3	3
	tRNA	40	40	42	40	41
	Plasmids	0	0	0	0	0

*The following formula was used to calculate genome coverage = read length × number of reads/genome length.

**Figure 4:**
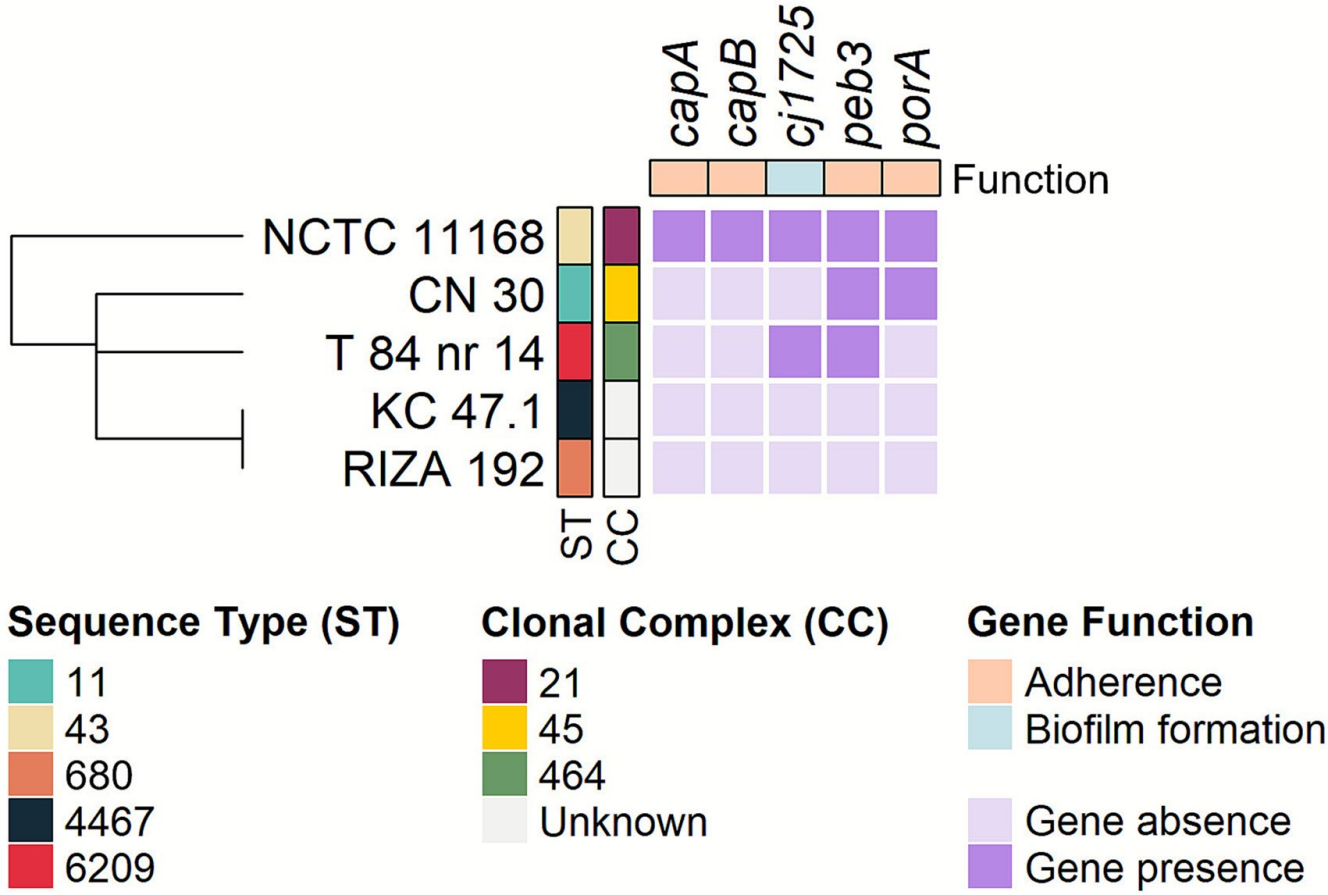
Presence/absence of adherence and biofilm-associated genes showing different occurrence in the *C. jejuni* strains. Sequence Types (ST) and Clonal Complexes (CC) are shown in colored boxes for each strain. The presence of a gene is indicated with dark purple color, while the absence is shown using light purple color. The dendrogram in the left represents the similarity among strains based on the gene/presence pattern and was generated according to the result of the hierarchical clustering using the complete linkage method on the Euclidean distance matrix. The heatmap was created using the ComplexHeatmap v.2.20.0 package (Gu et al., 2016) of the R software v.4.4.0 (R Core Team, 2024).

Among the 30 *C. jejuni* genes associated with adherence (*n* = 14) and biofilm formation (*n* = 16) that were examined (Supplementary Table 1), variations in their occurrence were limited to five genes, including the adherence genes *capA*, *capB*, *peb3*, and *porA*, as well as the *cj1725* gene, which is involved in biofilm formation (Figure 4). Only strain NCTC 11168 harbored all of them, as expected. Strain CN30 carried as well *peb3* and *porA*, while strain T 84 nr 14 also possessed *cj1725* and *peb3*. In contrast, all five genes were absent in strains KC 47.1 and RIZA 192. The remaining 25 adherence and biofilm formation genes were present in all isolates. Based on their adherence and biofilm formation gene profiles, strains KC 47.1 and RIZA 192 clustered together and were more closely related to strains CN 30 and T 84 nr 14, which formed a distinct cluster, than to strain NCTC 11168, which formed a separate group.

All strain carried a gene conferring resistance to *β*-lactams, as well as the multidrug efflux pump genes *cmeABC* and *cmeDEF*, together with their regulator *cmeR* (Table 4). In contrast, TET resistance mediated by the *tetO* gene was detected only in strain T 84 nr 14, which also harbored the T86I point mutation in *gyrA*, conferring resistance to the fluoroquinolones CIP and NAL. Additionally, this strain harbored the 50S rRNA L22 A103V mutation, that is suggested to be potentially associated with resistance to macrolides, including AZM and ERY. Furthermore, none of the strains carried resistance determinants for AZM, ERY, or GEN.

**Table 4:** Antibiotic resistance determinants identified in the *C. jejuni* strains tested in this study.

Strain	ST/CC	Resistance profile	Resistance determinants
CN 30	ST-11/CC45	β-LAC + MEP	*bla_OXA_-447* + *cmeABC*, *cmeDEF*, *cmeR*
KC 47.1	ST-4467	β-LAC + MEP	*bla_OXA_-447* + *cmeABC*, *cmeDEF*, *cmeR*
NCTC 11168	ST-43/CC21	β-LAC + MEP	*bla_OXA_-450* + *cmeABC*, *cmeDEF*, *cmeR*
RIZA 192	ST-680	β-LAC + MEP	*bla_OXA_-447* + *cmeABC*, *cmeDEF*, *cmeR*
T 84 nr 14	ST-6209/CC464	β-LAC + (F)QUI + MAC + TET + MEP	*bla_OXA_-450* + *gyrA* p.T86I + 50S rRNA L22 p.A103V + *tetO* + *cmeABC*, *cmeDEF*, *cmeR*

All the genes reported were found in at least the CARD database (McArthur et al., 2013), except *cmeDEF* that was only present in the Megares database (Lakin et al., 2017). ST/CC, Sequence Type/Clonal Complex; β-LAC, beta-lactams; (F)QUI, (fluoro)quinolones; MAC, macrolides; TET, tetracycline; MEP, multidrug efflux pump.

## 4 Discussion

In agreement with recent studies demonstrating the co-occurrence of microplastics (MPs) and *Campylobacter* spp. (Kolenc et al., 2024; Lo et al., 2025), the present study demonstrates that *Campylobacter jejuni* is capable of forming biofilms on MPs in a time-dependent and strain-specific manner. This is consistent with previous research on larger (polystyrene) plastic surfaces (Reeser et al., 2007; Teh et al., 2014; Ortega-Sanz et al., 2024). The findings indicate that rate and extent of biofilm development on MPs vary between *C. jejuni* strains and change differently over time, with strains reaching peak biofilm formation at different time points. While strain NCTC 11168 (ST-43/CC21) increased sessile cell density with longer incubation times, KC 47.1 (ST-4467) exhibited a decline in biofilm formation, similar to the reduction observed in *E. coli* on MPs (Ballesté et al., 2024). In contrast, other strains (ST-11/CC45, ST-680, and ST-6209/CC464) reached peak biofilm levels at intermediate incubation times. This observation contrast with the gradual decrease in biofilm formation reported for *E. coli* on MPs (∼1.8 log CFU/pellet at 48 h; average surface area of the plastic pellet = 77 mm^2^) (Ballesté et al., 2024). Therefore, MPs can serve as more stable reservoirs for *C. jejuni* than other foodborne pathogens, enhancing its environmental persistence and transmission through the food chain, thereby representing an added public health risk.

In this study, *C. jejuni* NCTC 11186 exhibited the strongest MP-associated biofilm formation overall, despite the widely reported observation that this strain generally forms relatively weak biofilms compared to other strains on larger (polystyrene) surfaces (Turonova et al., 2015; Araújo et al., 2021). This suggests that *C. jejuni* cell densities on MPs could exceed 5.8 log CFU/MP, and potentially even higher at lower inoculum concentrations (Araújo et al., 2021). Nonetheless, host-generalist strains of CC21 generally form more robust biofilms compared to host-specific strains (CC464) and sporadic strains (Nennig et al., 2022; Ortega-Sanz et al., 2024). Notably, biofilm formation on PET MPs appears to be substantially lower than on larger polystyrene surfaces, where the strain NCTC 11168 can reach approximately 6.77 ± 0.40 log CFU/well (surface area of the base = 32 mm^2^) on 96-well microtiter plates after incubation at 42 °C for 24 h (Klančnik et al., 2021). These observations highlight the critical influence of surface type, surface size and incubation conditions on *C. jejuni* biofilm development, suggesting that PET MPs may limit cell adhesion and proliferation compared to conventional laboratory plastics. In this regard, characterizing the surface roughness and hydrophobicity of MPs could provide additional insights into how plastic surface properties influence *C. jejuni* adherence. Moreover, in our study, certain strains also displayed biofilm formation behaviours that were consistent with their planktonic growth patterns (Ballesté et al., 2024). This suggests that viability of planktonic *C. jejuni* cells in liquid medium can influence biofilm development on MPs. However, notable fluctuations in the growth of *C. jejuni* culture have not been previously reported. Additionally, strains KC 47.1 and RIZA, which formed weaker biofilms on MPs, had larger genomes (1.8 Mbp) than strains with stronger biofilm-forming capabilities (1.6–1.7 Mbp). Reduced genome size has been associated with adaptive advantages in new environments, including faster growth rates and higher cell densities (LeBlanc and Charles, 2022). This genomic factor could also potentially influence the ability of *C. jejuni* to colonize MPs and may partly help explain the markedly reduced biofilm formation observed in strain KC 47.1. As all strains developed biofilms under identical conditions, differences in cell density and biofilm dynamics on MPs, as well as growth patterns, seem to result from a combination of genome size, specific genomic traits, and variable tolerance to environmental stresses such as pH, oxygen, and temperature.

The presence of adherence genes, rather than biofilm-associated genes, appears to determine the ability of *C. jejuni* to form biofilms on MPs, as 28.6% of adherence genes (4/14) showed variations in occurrence compared with only 6.3% (1/16) of biofilm-associated genes. The gene *peb3* may be critical for enhanced biofilm formation on MPs, as it was found exclusively in strains NCTC 11168, T 84 nr 14, and CN 30, which showed the highest biofilm levels throughout the incubation period. Similarly, the lack of *peb3* in strains KC 47.1 and RIZA 192 might indicate diminished biofilm formation capacity on MPs when absent. In *C. jejuni*, the PEB3 protein functions as both a cell surface adhesin and a periplasmic phosphate transporter, facilitating bacterial attachment to host cells and the uptake of essential molecules like 3-phosphoglycerate (Rangarajan et al., 2007). Similarly, the presence of *capA* and *capB* seemed to be associated with increased biofilm formation in strain NCTC 11168 at both early (24 h) and late (72 h) incubation times. In *C. jejuni*, CapA is an autotransporter protein that contributes to its ability to adhere to human epithelial cells and colonize the chicken gut (Ashgar et al., 2007). The function of CapB is less understood, with some studies failing to detect its expression and others suggesting it might act as a putative adhesin (Ashgar et al., 2007; Rubinchik et al., 2012). Taken together, these findings suggest that *peb3*, *capA*, and *capB* may play key roles in promoting elevated biofilm formation on MPs. In addition, the *porA* and *cj1725* genes could also be mutually exclusive (i.e., when one is present, the other is absent), as observed in strains T 84 nr 14 and CN 30, suggesting that each may be dispensable for enhanced biofilm formation on MPs. Either gene could potentially compensate for the absence of the other, supporting comparable or even higher biofilm formation levels on MPs. The gene *porA* encodes the major outer-membrane protein (MOMP), which acts as an adhesin critical for host cell attachment and infection (Sabotič et al., 2023). The mechanism of the putative periplasmic protein Cj1725 remains uncharacterized. However, its overexpression in biofilm cells of *C. jejuni* NCTC 11168 suggests it may exhibit a similar role on MPs. Nonetheless, as genes likely act collectively rather than individually (Liu et al., 2021; Selvam et al., 2024), it is possible that other genes and their interaction with the environment also influenced the *C. jejuni* biofilm formation levels on MPs. Indeed, biofilm formation is a multifactorial process in which approximately 600 genes are differentially expressed during biofilm formation, involving multiple pathways, such as motility, chemotaxis, and stress responses (Bundurus et al., 2024). Notably, the clustering of the *C. jejuni* strains in the phylogenetic tree, based on genomic similarities calculated using ANI, closely mirrored their biofilm-forming capacities. This suggests that the genomic content of *C. jejuni* may predispose certain strains to form biofilms on MPs. Nevertheless, these associations between biofilm-related genes and biofilm formation were in the current study derived from a limited set of five *C. jejuni* strains, underscoring the preliminary nature of the conclusions.

The *C. jejuni* strains exhibited high antibiotic susceptibility, with only one strain (ST-6209/CC464) displaying multidrug resistance (MDR) to at least three antimicrobial classes. Resistance profiles for the genotypes observed in this study recently analyzed, including CC45 and CC464, reveal that their antibiotic phenotypes and resistance patterns are similar to recent isolates (Elhadidy et al., 2018; García-Sánchez et al., 2019; Cobo-Díaz et al., 2021). This indicates that selective pressures from antibiotic use may have shaped these genotypes in comparable ways. Moreover, the antibiotic resistance profiles of the *C. jejuni* strains were consistent with their genomic data, indicating that specific genetic determinants correspond to observed resistance phenotypes (Concha-Toloza et al., 2023). In strain T 84 nr 14 (ST-6209/CC464), resistance to ciprofloxacin (CIP) and nalidixic acid (NAL) was conferred by the T86I mutation in *gyrA*, while resistance to tetracycline (TET) was mediated by the *tetO* gene. However, the 50S rRNA L22 A103V mutation, previously suggested to be associated with macrolide resistance (Gibreel and Taylor, 2006), did not confer resistance to azithromycin or erythromycin in this strain. This agrees with previous studies showing that the presence of this mutation alone is not sufficient to confer resistance in *C. jejuni* to these macrolides (Hull et al., 2021; Garcia-Fernandez et al., 2024). To the best of our knowledge, this is the first study that reports an association of ST-11, ST-680, and ST-4467 with susceptibility to AZM, CIP, ERY, GEN, NAL, and TET, while resistance of ST-6209 to CIP, NAL and TET. Additionally, all the *C. jejuni* strains tested could potentially be resistant to *β*-lactams due to the presence of genes encoding β-lactamase enzymes (*bla_OXA_*). Interestingly, the greater apparent antibiotic susceptibility of bacterial cells detached from MP-associated biofilms might be partly explained by the effect of sonication and temperature, which are known to induce the release of antibiotic resistance genes (Ko and Bai, 2022). However, in our study, the resistance phenotype of *C. jejuni* biofilm-derived cells was maintained, although their overall antibiotic susceptibility increased. This indicates that, even though the antibiotic resistance genes were probably retained in the chromosome, the sonication process (and the accompanying rise in temperature) may have imposed physiological stress on the cells released from the biofilm, leading to diminished MIC values. Increased antibiotic resistance in biofilm-detached *E. coli* cells formed on MPs has been reported by Gross et al. (2025), who vortexed the particles for 1 min to release the biofilm on their surface, opposite to our study. In addition, a lower number of viable cells in biofilm lawns could have increased their apparent antibiotic susceptibility compared to their planktonic counterparts, without affecting their underlying antibiotic resistance phenotype. Even with confluent growth, variable initial bacterial turbidities may occasionally have a minor effect on the Etest MICs (within 1 log_2_ dilution) without modifying the antibiotic resistance phenotype (Chang et al., 2001; Yildirim et al., 2023). The bacterial concentrations of the overnight cultures used in this study, representing planktonic populations, ranged from 7.8 to 8.3 log CFU/mL (data not shown), whereas the population densities of biofilm-derived bacteria ranged from 3.9 to 5.8 log CFU/mL. This corresponds to a variation in the viable cell density of the confluent bacterial lawns of approximately 2.5–3.9 log units (i.e., differences on the order of hundreds- to thousands-fold), which may have influenced the observed MIC values and potentially resulted in the apparent increase in antimicrobial susceptibility of biofilm-derived populations. Although research on the relationship between MPs and *C. jejuni* antibiotic resistance is still limited, it is well established that biofilms can substantially enhance the pathogen’s resistance compared to its planktonic state, with increases of up to 32-fold (Malik et al., 2017). Therefore, the findings of this study offer a valuable foundation, while also highlighting the need for further research of the full extent of these effects. This includes the development of alternative approaches that do not rely on strong shear forces, such as sonication, while still effectively assessing antibiotic resistance in weak biofilm-forming strains, in low-density biofilms, or directly in the biofilm lifestyle for minimal biofilm inhibitory concentration (MBIC) determination.

In conclusion, MPs pose a potential threat to food safety by facilitating *C. jejuni* contamination through biofilm formation, which may result in longer persistence of the pathogen in the environment. However, the capacity of *C. jejuni* to form biofilms on MPs varies between strains and fluctuates differently over time. Moreover, preliminary evidence suggests a limited role of MPs in promoting antibiotic resistance in *C. jejuni*, yet the development of appropriate methods is still necessary to accurately evaluate their impact on antimicrobial susceptibility.

## Data Availability

The datasets presented in this study can be found in online repositories. The names of the repository/repositories and accession number(s) can be found at: https://www.ebi.ac.uk/ena, PRJEB98259.

## Footnotes

1 http://www.bioinformatics.babraham.ac.uk/projects/fastqc.

2 https://pubmlst.org/organisms/campylobacter-jejunicoli, accessed September 18, 2025.

3 https://zenodo.org/records/14075401.

4 https://github.com/tseemann/abricate, accessed September 19, 2022.

5 https://www.ncbi.nlm.nih.gov/bioproject/PRJNA313047, accessed September 19, 2022.
