# Supplementary material for: Role of microplastics in the survival and antimicrobial susceptibility of Campylobacter jejuni
Source: Front Microbiol. 2026 Jan 5;16:1717297. doi: 10.3389/fmicb.2025.1717297 (PMC12813274; doi:10.3389/fmicb.2025.1717297)
Supplement: Supplementary file 1 [file Table_1.DOCX]

Supplementary Material

# Supplementary Tables

**Supplementary Table 1. List of adherence and biofilm-associated genes screened in the *C. jejuni* strains of study.**

| **Gene** | **Locus tag*** | **RefSeq gene ID** | **Gene location (bp)*** | **Gene length (bp)** | **Virulence Factor Category** |
| --- | --- | --- | --- | --- | --- |
| *clpP* | cj0192c | 906014 | c(187,535-188,119) | 585 | Biofilm formation |
| *frr* | cj0234c | 904561 | c(217,475-218,038) | 564 | Biofilm formation |
| *ilvE* | cj0269c | 904594 | c(247,656-248,570) | 915 | Adherence |
| *peb3* | cj0289c | 904613 | c(266622-267374) | 753 | Adherence |
| *ndk* | cj0332c | 904656 | c(301,366- 301,779) | 414 | Biofilm formation |
| *ribH* | cj0383c | 904706 | c(349,139-349,603) | 465 | Biofilm formation |
| *cj0391c* | cj0391c | 904714 | c(358,017-358,652) | 636 | Biofilm formation |
| *yceI* | cj0420 | 904744 | 385,195-385,767 | 573 | Biofilm formation |
| *sucD* | cj0534 | 904862 | 498,354-499,223 | 870 | Biofilm formation |
| *tlyA* | cj0588 | 904912 | 548,257-549,018 | 762 | Adherence |
| *peb4* | cj0596 | 904921 | 553,232-554,053 | 822 | Adherence |
| *capA* | cj0628/9 | 904958 | 587,868-591,303 | 3,436 | Adherence |
| *hiuH* | cj0715 | 905032 | 669,395-669,808 | 414 | Biofilm formation |
| *metQ* | cj0772c | 905082 | c(721,828-722,601) | 774 | Biofilm formation |
| *peb2* | cj0778 | 905087 | 730,569-731,306 | 738 | Adherence |
| *peb1A* | cj0921c | 906008 | c(857,098-857,877) | 780 | Adherence |
| *purL* | cj0955c | 904957 | c(894,780-896,966) | 2,187 | Biofilm formation |
| *jlpA* | cj0983 | 905274 | 916,626-917,744 | 1,119 | Adherence |
| *groES* | cj1220 | 905510 | 1,149,194-1,149,454 | 261 | Biofilm formation |
| *groEL* | cj1221 | 905511 | 1,149,475-1,151,112 | 1,638 | Biofilm formation |
| *porA* | cj1259 | 905550 | 1,189,121-1,190,395 | 1,275 | Adherence |
| *flpA* | cj1279c | 905570 | c(1,211,677-1,212,912) | 1,236 | Adherence |
| *pgp1* | cj1345c | 905637 | c(1,277,455-1,278,849) | 1,395 | Adherence |
| *pldA* | cj1351 | 905643 | 1,282,933-1,283,922 | 990 | Adherence |
| *cadF* | cj1478c | 905765 | c(1,413,913-1,414,872) | 960 | Adherence |
| *fdhA* | cj1511c | 905795 | c(1,446,341-1,449,145) | 2,805 | Biofilm formation |
| *cj1626c* | cj1626c | 905898 | c(1,554,644-1,555,060) | 417 | Biofilm formation |
| *cj1631c* | cj1631c | 905904 | c(1,557,525-1,558,394) | 870 | Biofilm formation |
| *capB* | cj1677/8 | 905952 | 1,597,008-1,600,372 | 3,365 | Adherence |
| *cj1725* | cj1725 | 905202 | 1,634,839-1,635,438 | 600 | Biofilm formation |

*Genes located on the complementary DNA strand are indicated as “c”. All gene sequences were retrieved from the genome of the *Campylobacter jejuni* subsp. *jejuni* strain NCTC 11168 (NC_002163.1).

**Supplementary Table 2. Statistical results for the quantification of sessile and planktonic bacteria of *Campylobacter jejuni* during biofilm formation on microplastics at 37 °C under microaerobic conditions.**

| **Effect** | **Factor** | **Df** | **Sessile bacteria*** | **Planktonic bacteria (MP)^†^** | **Planktonic bacteria (no-MP)^†^** |
| --- | --- | --- | --- | --- | --- |
| Principal | Strain | 4 | F = 26.9  *p* = 5.659e-10 | F = 66.5  *p* = 9.207e-15 | F = 56.2  *p* = 9.083e-14 |
|  | Incubation time | 2 | F = 14.8  *p* = 2.624e-05 | F = 3.4  *p* = 0.044 | F = 3.1  *p* = 0.057 |
| Interaction | Strain-incubation time | 8 | F = 10.4  *p* = 3.915e-07 | F = 7.1  *p* = 2.637e-05 | F = 4.2  *p* = 0.002 |

*, non-parametric Aligned Rank Transform (ART) ANOVA was applied. ^†^, parametric ANOVA was applied. Df, degrees of freedom. F, F-statistic. *p*, p-value.

**Supplementary Table 3. Quantification of sessile (log CFU/MP) and planktonic (log CFU/mL) cells of *Campylobacter jejuni* during biofilm formation on microplastics at 37 °C under microaerobic conditions.**

|  |  | **Incubation time (h)** | | |
| --- | --- | --- | --- | --- |
| **Lifestyle** | **Strain** | **24** | **48** | **72** |
| **Biofilm*** | **CN 30** | 4.38 ± 0.56 ^def^ | 4.67 ± 0.36 ^ef^ | 4.39 ± 0.40 ^def^ |
|  | **KC 47.1** | 3.42 ± 0.23 ^cd^ | 1.09 ± 0.24 ^a^ | 2.20 ± 0.33 ^ab^ |
|  | **NCTC 11168** | 4.60 ± 0.34 ^ef^ | 4.29 ± 0.17 ^def^ | 5.78 ± 0.20 ^g^ |
|  | **RIZA 192** | 2.99 ± 0.10 ^bc^ | 4.31 ± 0.18 ^def^ | 3.94 ± 0.17 ^cde^ |
|  | **T 84 nr 14** | 4.26 ± 0.76 ^def^ | 4.99 ± 0.67 ^efg^ | 5.14 ± 0.21 ^fg^ |
| **Planktonic (MP)^†^** | **CN 30** | 7.93 ± 0.33 ^def^ | 7.37 ± 0.57 ^cde^ | 7.02 ± 0.46 ^bcd^ |
|  | **KC 47.1** | 6.17 ± 0.49 ^bc^ | 4.22 ± 0.26 ^a^ | 5.74 ± 0.48 ^b^ |
|  | **NCTC 11168** | 7.86 ± 0.19 ^def^ | 8.22 ± 0.76 ^def^ | 8.96 ± 0.20 ^f^ |
|  | **RIZA 192** | 5.78 ± 0.11 ^b^ | 7.13 ± 0.81 ^bcde^ | 6.14 ± 0.33 ^bc^ |
|  | **T 84 nr 14** | 7.60 ± 0.15 ^de^ | 7.98 ± 0.62 ^def^ | 8.48 ± 0.23 ^ef^ |
| **Planktonic (no-MP)^†^** | **CN 30** | 8.10 ± 0.07 ^efg^ | 7.42 ± 0.37 ^cdef^ | 7.44 ± 0.35 ^cdef^ |
|  | **KC 47.1** | 6.29 ± 0.41 ^bcd^ | 4.57 ± 0.66 ^a^ | 5.43 ± 1.07 ^ab^ |
|  | **NCTC 11168** | 7.99 ± 0.41 ^efg^ | 8.37 ± 0.68 ^fg^ | 8.94 ± 0.31 ^g^ |
|  | **RIZA 192** | 6.06 ± 0.60 ^abc^ | 7.11 ± 0.36 ^bcdef^ | 6.70 ± 0.02 ^bcde^ |
|  | **T 84 nr 14** | 7.65 ± 0.05 ^defg^ | 7.97 ± 0.73 ^efg^ | 8.70 ± 0.12 ^fg^ |

Non-parametric Aligned Rank Transform (ART) ANOVA (*) or parametric ANOVA (^†^) followed by post-hoc pairwise comparisons of estimated marginal means with Tukey correction for multiple comparisons were performed. Different letters indicate statistically significant differences between strain × incubation time combinations for each lifestyle (*p* ≤ 0.05).

**Supplementary Table 4. Replicate values of all minimum inhibitory concentration (MIC) measurements (in mg/L).**

| **Lifestyle** | **Strain** | **Replicate** | **AZM** | **CIP** | **ERY** | **GEN** | **NAL** | **TET** |
| --- | --- | --- | --- | --- | --- | --- | --- | --- |
| Planktonic | CN 30 | 1 | 0.125 | 0.125 | 1 | 0.75 | 3 | 0.25 |
|  |  | 2 | 0.125 | 0.125 | 1 | 0.75 | 3 | 0.25 |
|  |  | 3 | 0.094 | 0.125 | 0.75 | 0.75 | 2 | 0.25 |
|  | KC 47.1 | 1 | 0.094 | 0.064 | 0.75 | 0.5 | 3 | 0.25 |
|  |  | 2 | 0.094 | 0.064 | 0.5 | 0.5 | 2 | 0.25 |
|  |  | 3 | 0.094 | 0.064 | 0.75 | 0.5 | 3 | 0.25 |
|  | NCTC 11168 | 1 | 0.064 | 0.094 | 0.75 | 0.75 | 4 | 0.094 |
|  |  | 2 | 0.094 | 0.064 | 0.75 | 1 | 3 | 0.125 |
|  |  | 3 | 0.094 | 0.094 | 1 | 0.75 | 3 | 0.125 |
|  | RIZA 192 | 1 | 0.064 | 0.064 | 0.5 | 0.5 | 1.5 | 0.19 |
|  |  | 2 | 0.047 | 0.047 | 0.5 | 0.5 | 1 | 0.19 |
|  |  | 3 | 0.064 | 0.047 | 0.38 | 0.5 | 1 | 0.125 |
|  | T 84 nr 14 | 1 | 0.094 | 6 | 0.75 | 0.38 | >256 | 64 |
|  |  | 2 | 0.125 | 6 | 1 | 0.25 | >256 | 64 |
|  |  | 3 | 0.125 | 6 | 0.75 | 0.38 | >256 | 64 |
| Detached biofilm | CN 30 | 1 | 0.032 | 0.032 | 0.25 | 0.38 | 1.5 | 0.094 |
|  |  | 2 | 0.023 | 0.032 | 0.19 | 0.38 | 1 | 0.064 |
|  |  | 3 | 0.023 | 0.023 | 0.19 | 0.38 | 1.5 | 0.064 |
|  | NCTC 11168 | 1 | 0.064 | 0.064 | 0.5 | 0.38 | 2 | 0.064 |
|  |  | 2 | 0.064 | 0.064 | 0.5 | 0.38 | 2 | 0.064 |
|  |  | 3 | 0.064 | 0.064 | 0.5 | 0.38 | 2 | 0.064 |
|  | RIZA 192 | 1 | 0.016 | 0.002 | 0.125 | 0.094 | 0.75 | 0.094 |
|  |  | 2 | 0.016 | 0.002 | 0.125 | 0.094 | 0.5 | 0.094 |
|  |  | 3 | 0.023 | 0.002 | 0.125 | 0.125 | 0.5 | 0.064 |
|  | T 84 nr 14 | 1 | 0.064 | 4 | 0.25 | 0.125 | >256 | 32 |
|  |  | 2 | 0.064 | 3 | 0.19 | 0.125 | >256 | 32 |
|  |  | 3 | 0.064 | 4 | 0.19 | 0.125 | >256 | 64 |

AZM, azithromycin. CIP, ciprofloxacin. ERY, erythromycin. GEN, gentamicin. NAL, nalidixic acid. TET, tetracycline.
